# Early Caloric Adequacy and Hospital Length of Stay in Children Younger than Two Years Hospitalized with Bronchiolitis

**DOI:** 10.3390/nu18182989

**Published:** 2026-09-12

**Authors:** Ana Barrés-Fernández, José Vicente Arcos-Machancoses, Silvia Castillo-Corullón, Susana Ferrando-Monleón, Caterina Calderon, Cecilia Martínez-Costa

**Affiliations:** 1 Department of Pediatrics, Hospital Clínic Universitari de València, 46010 Valencia, Spain; arjovi@uv.es (J.V.A.-M.); castillo_sil@gva.es (S.C.-C.); su.ferrandom@comv.es (S.F.-M.); cecilia.martinez@uv.es (C.M.-C.); 2 INCLIVA Health Research Institute, 46010 Valencia, Spain; 3 Department of Pediatrics, Obstetrics and Gynecology, Faculty of Medicine and Dentistry, University of Valencia, 46010 Valencia, Spain; 4 Department of Clinical Psychology and Psychobiology, University of Barcelona, 08035 Barcelona, Spain; ccalderon@ub.edu

**Keywords:** bronchiolitis, caloric intake, length of stay, nutritional status

## Abstract

**Background/Objectives**: Feeding difficulties are common during bronchiolitis hospitalization and may lead to reduced caloric intake. Evidence evaluating the relationship between caloric adequacy and hospital length of stay (LOS) remains limited. We aimed to characterize daily caloric adequacy during bronchiolitis hospitalization and evaluate its relationship with LOS. **Methods**: An ambispective cohort study included children younger than 2 years hospitalized with bronchiolitis between 2013 and 2023 at a tertiary hospital in Valencia, Spain. Caloric adequacy was calculated as the percentage of WHO/FAO estimated caloric requirements achieved, including all nutritional and intravenous caloric sources. Associations with LOS were evaluated using correlation and multivariable regression analyses. **Results**: A total of 613 patients were included, with caloric intake data available for 607. Median LOS was 5 days (IQR 3–7). Lower caloric adequacy during the first days of hospitalization was associated with longer LOS. Hospitalization day 2 showed the strongest early association with LOS (ρ = −0.230; *p* <0.001). After multivariable adjustment, each 10-percentage-point increase in day-2 caloric adequacy was associated with a 3.32% shorter LOS (95% CI, 1.74–4.87%; *p* < 0.001); subgroup associations were mainly observed in children younger than 12 months and were strongest in those with moderate acute malnutrition. Patients with longer LOS showed delayed achievement of ≥80% caloric adequacy, particularly those with LOS ≥ 9 days (day 6). **Conclusions**: Early caloric adequacy was associated with LOS in children hospitalized with bronchiolitis. Failure to achieve ≥80% within the first 48 h may serve as an early marker of a slower clinical course.

## 1. Introduction

Acute bronchiolitis is the leading cause of hospitalization for lower respiratory tract infection in infants and young children worldwide [1,2]. Feeding difficulties are common because of tachypnoea, increased work of breathing, fatigue, and vomiting, frequently resulting in reduced caloric intake during the acute phase of illness [1,3,4,5]. Similar feeding difficulties have also been reported in other acute pediatric respiratory illnesses requiring hospitalization, where respiratory distress may temporarily impair oral intake and increase the need for nutritional support [6,7].

Because bronchiolitis predominantly affects young infants with high energy requirements and limited nutritional reserves, even short periods of inadequate caloric intake may rapidly lead to a negative energy balance. In addition, acute respiratory infections are associated with increased metabolic demands, potentially compromising nutritional recovery and clinical evolution [8,9,10,11]. Consequently, early nutritional assessment and timely nutritional support have become key components of supportive management in hospitalized infants.

Despite the clinical importance of nutritional support, evidence evaluating the relationship between caloric adequacy and clinical outcomes in bronchiolitis remains limited. Previous studies have suggested that lower caloric intake during the first days of hospitalization is associated with prolonged hospital length of stay (LOS) and delayed nutritional recovery [12,13]. However, accurately quantifying caloric intake in hospitalized infants remains challenging, particularly among breastfed infants and those receiving complementary feeding. A more comprehensive assessment of caloric intake may therefore improve our understanding of the relationship between early nutritional achievement and clinical outcomes.

Accordingly, the aim of the present study was to characterize daily caloric adequacy throughout hospitalization in children younger than two years admitted with bronchiolitis, using a previously published probabilistic breastfeeding model developed by our group [14], and to examine its association with hospital LOS, both overall and across clinically relevant patient subgroups.

## 2. Materials and Methods

### 2.1. Study Design and Population

An ambispective cohort study was conducted at a tertiary-care hospital in Valencia, Spain, between January 2013 and December 2023. The cohort comprised a retrospective phase from January 2013 to June 2017 and a prospective phase from July 2017 to December 2023. The transition to prospective data collection coincided with the implementation of a nutritional protocol for children hospitalized with bronchiolitis in July 2017. The study included children younger than 2 years hospitalized with acute bronchiolitis. Bronchiolitis was diagnosed clinically as a first episode of acute respiratory distress associated with signs of viral upper respiratory tract infection in children younger than 24 months [15]. Bronchiolitis severity was assessed at Emergency Department presentation using the modified Wood–Downes–Ferrés (WDFM) score and the Hospital Sant Joan de Déu (HSJD) bronchiolitis severity score. The HSJD score incorporates age-specific criteria in the assessment of disease severity [16,17,18]. Following initial measures, including nasal saline irrigation and suctioning of respiratory secretions, patients were clinically reassessed. Only patients with mild-to-moderate bronchiolitis according to at least one of the two severity scores were included.

Patients requiring immediate admission to the pediatric intensive care unit (PICU), those with severe chronic underlying diseases, major congenital anomalies, neuromuscular disorders, severe congenital heart disease, chronic respiratory disease unrelated to prematurity, or incomplete nutritional records were excluded.

Medical records were identified and reviewed using discharge diagnoses coded according to the International Classification of Diseases, 11th Revision (ICD-11), including acute bronchiolitis and respiratory failure. Data were independently verified by a statistician.

### 2.2. Daily Caloric Adequacy Assessment

Daily caloric intake was defined as the total number of kilocalories consumed or estimated during each hospitalization day and was expressed as kcal/kg/day. Each hospitalization day was calculated from the exact admission time to the following 24-h interval according to nursing documentation.

Daily caloric goals were estimated according to age-based World Health Organization/Food and Agriculture Organization (WHO/FAO) recommendations [19,20].

Daily caloric adequacy was calculated as the ratio between total daily caloric intake and the estimated daily caloric requirement, multiplied by 100: Caloric adequacy (%) = (total daily caloric intake/estimated daily caloric requirement) × 100.

For exploratory descriptive analyses, a prespecified threshold of ≥80% of estimated caloric requirements was used to characterize the trajectory of caloric adequacy during hospitalization. This cut-off was selected as a pragmatic marker of substantial caloric goal attainment, for comparability with prior literature, and was not intended to represent a validated therapeutic target for bronchiolitis.

### 2.3. Caloric Intake Estimation

Daily nutritional intake, including breastfeeding episodes, formula volume, and complementary food intake, was routinely documented by nursing staff as part of standard clinical care throughout the entire study period. This documentation practice was already in place before the implementation of the nutritional protocol in 2017 and remained unchanged thereafter. Total daily caloric intake included calories derived from formula feeding, breastfeeding, complementary feeding, and intravenous fluid therapy.

Formula feeding caloric intake was calculated using standardized caloric density values (0.6–0.7 kcal/mL) [21].

Breast milk caloric intake was estimated using a previously published probabilistic model developed for hospitalized infants. The model incorporated variability in feeding frequency, milk volume per feeding, caloric density, and infant anthropometric characteristics through Monte Carlo simulations, providing age-stratified estimations of caloric intake from breastfeeding. Three caloric intake scenarios were generated from the simulated distributions: low estimate (25th percentile), medium estimate (50th percentile), and high estimate (75th percentile) [14]. The medium estimate was used as the reference scenario for all primary analyses.

Caloric intake from complementary feeding was estimated using standardized nutritional information provided by the hospital dietetics unit, including pureed foods, solid foods and cereal preparations. The detailed nutritional composition of these foods is provided in Appendix A.

Intravenous fluid caloric intake was estimated according to the glucose-containing maintenance fluids routinely administered in the infant ward.

Only data from complete 24-h hospitalization days were included in the analysis; partial days, including the day of discharge when incomplete, were excluded.

### 2.4. Outcomes

The primary outcome was the association between early caloric adequacy and hospital LOS. Secondary outcomes included the characterization of daily caloric adequacy throughout hospitalization and exploratory analyses across clinically relevant patient subgroups, including age, sex, home feeding type and nutritional status at admission.

Nutritional status at admission was assessed using weight-for-length z-scores according to WHO standards and categorized as severe acute malnutrition (z-score < −3 standard deviations [SD]), moderate acute malnutrition (z-score −3 to −2 SD), normal nutritional status (z-score −2 to +1 SD), risk of overweight (z-score +1 to +2 SD), overweight (z-score +2 to +3 SD), and obesity (z-score > +3 SD) [22].

Hospitalization day 2 (D2) was prespecified as the key time point for analyses of early caloric adequacy based on previous bronchiolitis studies that identified caloric intake on D2 as a nutritional marker associated with hospital LOS. This time point was also considered clinically relevant because it provides an opportunity to identify patients with inadequate caloric intake [12,13]. Daily caloric adequacy beyond the prespecified early time point was analysed descriptively among patients who remained hospitalized on each corresponding day. Because availability of caloric intake data on later hospitalization days was conditional on continued hospitalization, these analyses were not interpreted as longitudinal associations with LOS.

In anticipation of PICU admission being a consequence of clinical evolution likely occurring after the D2 exposure window (median onset day 3 in the retrospective sample), it was not included as a covariate in the primary multivariable model, to avoid conditioning on a post-exposure event.

### 2.5. Statistical Analysis

The distribution of continuous variables was assessed using the Shapiro–Wilk test. Continuous variables were expressed as mean ± standard deviation (SD) or median with interquartile range (IQR), as appropriate.

Categorical variables were expressed as frequencies and percentages. Comparisons between groups were performed using the Mann–Whitney U test or the Kruskal–Wallis test with Bonferroni correction for post hoc pairwise comparisons, as appropriate.

Correlations between caloric adequacy and hospital LOS were evaluated using Spearman correlation coefficients (ρ). Pearson correlation coefficients (r) were also calculated for exploratory analyses.

The association between D2 caloric adequacy and LOS was additionally assessed with multivariable linear regression on log-transformed LOS (because raw LOS was right-skewed; skewness ≈ 1.3), adjusting for age at admission, prematurity, feeding refusal, weight/length z-score, feeding type, and baseline severity. Effect sizes are expressed as the percentage change in LOS per 10-percentage-point increase in D2 adequacy. Two severity instruments (WDFM and HSJD) were used in a sensitivity analysis. As a secondary, descriptive analysis, D2 caloric intake adequacy was also dichotomized at the 80% threshold (<80% vs. ≥80%); this cut-point is not proposed as a validated clinical target.

To address temporal overlap between the D2 exposure window and the LOS outcome window, a landmark analysis was performed with the landmark set at the end of D2 (48 h after admission). Because D2 caloric adequacy was calculated only from complete 24-h hospitalization days, patients discharged before completion of the D2 exposure window were not eligible for the landmark analysis. Among patients remaining hospitalized after the 48-h landmark, log-transformed time from the landmark to discharge was modelled as a function of D2 caloric adequacy, adjusted for the same covariates as the primary model.

Given the ambispective design, baseline characteristics, severity, D2 adequacy, D2 missingness, and LOS were compared between phases, and study phase was also included as a covariate in all multivariable models. As a further sensitivity analysis, the primary model was re-estimated after excluding admissions occurring during the pandemic trough in bronchiolitis presentations (March 2020–April 2021), a period of near-complete suppression of Respiratory Syncytial Virus circulation due to non-pharmaceutical interventions.

Statistical significance was defined as *p* < 0.05. Analyses were performed using SPSS version 27.0 (IBM Corp., Armonk, NY, USA) [23].

## 3. Results

### 3.1. Patient Characteristics

A total of 613 children hospitalized with bronchiolitis were included in the study (Table 1). The cohort was predominantly composed of young infants, with a median age at admission of 71 days (IQR 34–124); 88% were younger than 6 months and 96% were younger than 12 months. At Emergency Department presentation, severe bronchiolitis was identified in 9% of patients according to the WDFM score and in 1% according to the HSJD score. Following initial supportive measures and clinical reassessment, all patients included in the study were classified as having mild-to-moderate bronchiolitis according to at least one of the two severity scores. Feeding refusal before admission was present in 75% of patients. Almost half of the cohort (48%) required hydration/nutritional support during hospitalization. Supplemental oxygen therapy was required in 378 patients (62%), while 28 (5%) received high-flow nasal cannula (HFNC) therapy. Among patients receiving oral or nasogastric enteral feeding during HFNC therapy, no aspiration events or feeding-related respiratory deterioration were documented, including when estimated caloric requirements were achieved. Four percent of patients required PICU admission. Median hospital LOS was 5 days (IQR 3–7).

### 3.2. Daily Caloric Adequacy According to Hospital LOS

**Figure 1:**
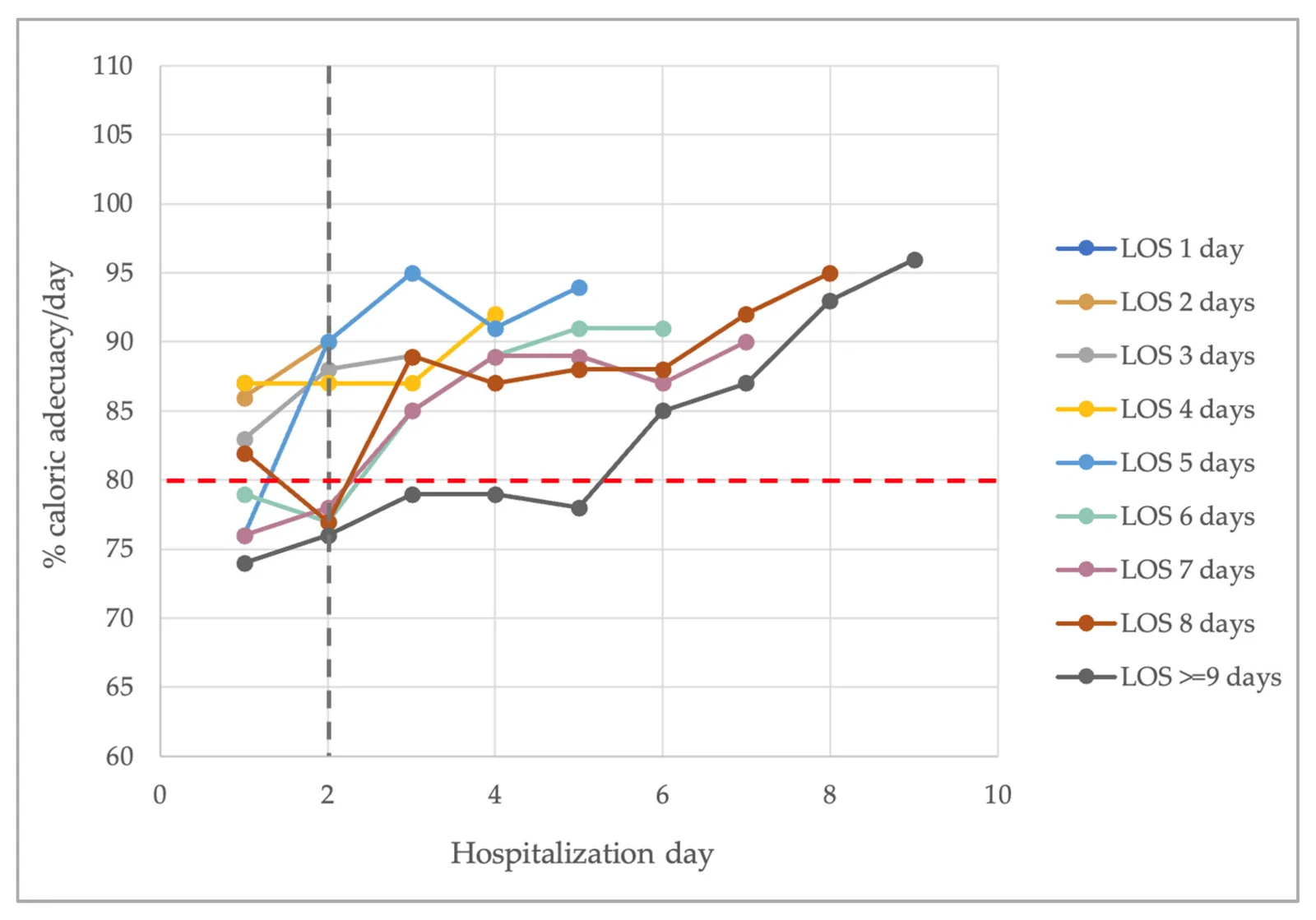
Daily caloric adequacy during hospitalization according to hospital LOS. Line graph showing the evolution of daily caloric adequacy (% caloric adequacy/day) during hospitalization according to hospital length of stay (LOS). Caloric adequacy was calculated using the median breastfeeding estimate (50th percentile) derived from the probabilistic breastfeeding model [14]. The horizontal dashed red line indicates the predefined threshold for early caloric adequacy (80% of estimated caloric requirements), whereas the vertical dashed dark grey line marks hospitalization day 2, the prespecified key time point for early caloric adequacy analyses.

Daily caloric adequacy data were available for 607 of the 613 patients included in the cohort. Daily caloric adequacy according to hospital LOS, using the medium breastfeeding estimate, is shown in Table 2 and Figure 1. Patients grouped according to longer LOS showed lower caloric adequacy during the first days of hospitalization and delayed achievement of caloric goals over time.

Patients with LOS > 6 days did not achieve ≥80% caloric adequacy until hospitalization day 3, whereas patients with LOS ≥ 9 days reached this threshold only by day 6.

Overall, failure to achieve ≥80% caloric adequacy during the first 48 h was consistently associated with longer hospital stays.

### 3.3. Relationship Between Early Caloric Adequacy and Hospital LOS

On day 1, median caloric adequacy was 80% (IQR 65–94), with a weak but significant correlation with LOS (ρ = −0.150; *p* = 0.001).

Hospitalization D2 showed the strongest early association between caloric adequacy and LOS. Median caloric adequacy increased to 86% (IQR 71–100), with a modest inverse correlation with LOS (ρ = −0.230; *p* < 0.001).

Exploratory day-specific correlations for subsequent hospitalization days are shown in Table 3. These analyses include only patients who remained hospitalized on each corresponding day and should therefore be interpreted descriptively rather than as longitudinal associations with LOS.

### 3.4. Multivariable Analysis

In a multivariable linear regression model adjusting for age, prematurity, feeding refusal, nutritional status, feeding type, baseline WDFM severity, and study phase, each 10-percentage-point increase in D2 caloric adequacy remained associated with a 3.32% shorter LOS (95% CI, −4.87 to −1.74%; *p* < 0.001) (Table 4; full covariate output in Table A1). This association was materially unchanged when baseline severity was measured with the HSJD instrument instead of WDFM (−3.38%; 95% CI, −4.94 to −1.79%; *p* < 0.001). In a secondary, descriptive analysis, failing to reach 80% of the D2 caloric target was associated with a 18.68% longer LOS compared with reaching ≥80% (95% CI, 10.75–26.77%; *p* < 0.001; Table 4; Table A1).

### 3.5. Landmark Analysis

To address temporal overlap between the D2 exposure window and the LOS outcome window, a landmark analysis was performed at the end of D2 (48 h after admission). Among patients remaining hospitalized after the 48-h landmark, each 10-percentage-point increase in D2 caloric adequacy remained associated with a shorter time from the landmark to discharge (−5.10%; 95% CI, −7.36 to −2.79%; *p* < 0.001; Table 4), suggesting that the observed association was not fully explained by temporal overlap between the exposure and outcome windows.

### 3.6. Ambispective Phase Comparison

After correction for multiple comparisons (Bonferroni, 11 tests), the retrospective (*n* = 255) and prospective (*n* = 358) phases differed significantly in age at admission, baseline WDFM severity, and D2 caloric adequacy (all *p* < 0.05, corrected; Table A2), supporting inclusion of study phase as an adjustment variable. Feeding-refusal severity differed at the uncorrected threshold (*p* = 0.010) but not after correction (*p* = 0.112) and is not interpreted as a phase difference. D2 missingness did not differ significantly between phases (0.0% vs. 0.8%; *p* = 0.38). LOS itself was similar between phases (median 5 vs. 5 days; *p* = 0.073). Excluding the 6 admissions occurring during the pandemic trough (March 2020–April 2021), all of which fell within the prospective phase, did not materially change the primary association (−3.1%; *p* < 0.001), suggesting that the observed association was not influenced by the pandemic period.

### 3.7. Subgroup Analyses

Subgroup analyses were performed using hospitalization D2 (*n* = 594), the prespecified key time point for early caloric adequacy analyses.

Results according to demographic and clinical subgroups are summarized in Table 5.

By age, significant inverse correlations were observed in infants aged <6 months (ρ = −0.147; *p* < 0.001) and 6–11 months (ρ = −0.269; *p* = 0.026), whereas no significant associations were identified in older age groups.

No differences in D2 caloric adequacy were observed according to sex; however, significant inverse correlations with LOS were found in both females (ρ = −0.183; *p* = 0.004) and males (ρ = −0.153; *p* = 0.003).

Breastfed infants achieved the highest median D2 caloric adequacy (87%, IQR 76–102), followed by mixed-fed (83%, IQR 70–102) and formula-fed infants (82%, IQR 67–97). Post hoc pairwise comparisons with Bonferroni correction showed significantly higher caloric adequacy in breastfed compared with formula-fed infants (*p* = 0.029). Significant inverse correlations with LOS were observed in both breastfeeding and formula-feeding groups.

No differences in caloric adequacy were observed according to nutritional status at admission. However, infants with moderate acute malnutrition showed the strongest inverse association between D2 caloric adequacy and LOS (ρ = −0.449; *p* = 0.002).

## 4. Discussion

The present study highlights the association between early caloric adequacy and clinical course during bronchiolitis hospitalization [12,24,25,26]. Patients with delayed achievement of caloric goals during the first days of hospitalization exhibited a less favourable nutritional trajectory and tended to have longer hospital stays. These findings are consistent with the limited evidence currently available and reinforce the importance of early caloric intake during hospitalization, even in patients requiring respiratory support such as HFNC, in whom nasogastric enteral nutrition may be required when adequate oral intake cannot be maintained. Nevertheless, these patients require closer monitoring for potential feeding-related complications, including feeding intolerance, vomiting, aspiration, or worsening respiratory compromise [24,25,26].

The first 48 h were particularly informative. Caloric adequacy progressively increased during hospitalization, but D2 showed the strongest early association with LOS, although the magnitude of the unadjusted correlation was modest. This time point may reflect the transition from initial clinical stabilization to feeding recovery [9,27,28] and is consistent with previous studies focusing nutritional assessment on the second hospitalization day [12]. Importantly, the association between D2 caloric adequacy and LOS persisted after multivariable adjustment and in the landmark analysis anchored at the end of D2, suggesting that it was not fully explained by baseline disease severity or by the temporal overlap between D2 caloric assessment and total LOS.

Beyond the early hospitalization period, the association between caloric adequacy and LOS remained negative in direction but progressively lost statistical significance at later time points. These analyses included progressively smaller and selected populations because caloric adequacy could only be assessed in patients who remained hospitalized on each corresponding day. Therefore, consistent with the literature highlighting the relevance of the early hospitalization period, our primary interpretation focuses on Days 1–2, whereas findings from later hospitalization days should be considered exploratory and descriptive [12,13].

One main methodological difference concerns the estimation of caloric intake from breastfeeding between our work and a previous study. Weisgerber et al. estimated breast milk intake using fixed assumptions regarding feeding frequency and caloric contribution according to age. Although pragmatic, this approach does not account for interindividual variability and assumes a homogeneous caloric contribution among breastfed infants [12]. In contrast, the present study used a previously published probabilistic model developed by our group incorporating variability in feeding frequency, milk volume, caloric density, and infant anthropometric characteristics, providing a more individualized estimation of breastfeeding intake [14].

Another strength of the present study was the inclusion of calories derived from complementary feeding, pureed foods, solid foods, cereal supplementation, and glucose-containing intravenous fluids. Previous studies did not account for these caloric sources [12], potentially underestimating total caloric intake, particularly in older infants who had already started complementary feeding. By incorporating all relevant caloric sources, the present analysis provides a more comprehensive assessment of nutritional intake during hospitalization.

The subgroup analyses also yielded relevant findings. The association between caloric adequacy and LOS remained significant only in children younger than 12 months, supporting the concept that early infancy may represent the period of greatest nutritional vulnerability during bronchiolitis. This observation is biologically plausible, since feeding during the first year of life depends predominantly on milk intake and relatively small reductions in intake may have a greater nutritional impact [29].

Another relevant finding was the stronger association observed among infants with moderate acute malnutrition at admission. Although this subgroup represented a small proportion of the cohort, these results suggest that pre-existing nutritional vulnerability may strengthen the association between inadequate intake and clinical course during acute illness [12]. A weaker but still significant association was also observed among children with normal nutritional status, suggesting that the association with early caloric adequacy may extend beyond patients with overt malnutrition. This observation is biologically plausible, as many infants with bronchiolitis already present feeding difficulties and reduced oral intake before hospitalization, potentially leading to an early nutritional deficit before admission. In our cohort, feeding refusal was reported in 75% of patients before hospitalization [12,13,30].

Breastfed infants achieved slightly higher caloric adequacy values than formula-fed children. However, this finding should be interpreted cautiously, as breast milk intake was estimated rather than directly measured. Nevertheless, significant associations between caloric adequacy and LOS were identified in both groups, suggesting that the association was observed across different feeding modalities.

A clinically relevant finding was that patients with prolonged hospitalizations more frequently failed to achieve at least 80% of estimated caloric requirements during the first 48 h of admission. In contrast, earlier attainment of this threshold was more common among patients with shorter LOS. Although exploratory, these findings suggest that failure to achieve early caloric adequacy may represent a marker of a slower clinical course. However, the ≥80% cut-off was not validated as a therapeutic target, and these findings should not be interpreted as evidence that increasing caloric delivery to achieve this threshold would reduce hospital LOS.

The present study included the largest cohort evaluating caloric adequacy in bronchiolitis reported to date and incorporated patients from ten bronchiolitis seasons. Furthermore, the comprehensive assessment of caloric intake, including all nutritional sources together with a probabilistic estimation of breastfeeding intake, represents an important methodological strength that may improve the accuracy of caloric adequacy assessment.

Several limitations should be acknowledged. First, this was a single-centre study, which may limit external validity. Second, despite using a probabilistic model, breast milk intake remained estimated rather than directly measured. Third, the observational design does not allow causal inference. Reduced caloric intake may represent both a contributing factor and a marker of disease severity, and residual confounding cannot be excluded. In particular, patients with more severe disease may tolerate lower caloric intake due to respiratory compromise, which may in turn prolong hospital stay (reverse causality). Additionally, unmeasured factors such as feeding practices, clinical management, and variability in nutritional support strategies may have influenced both caloric intake and outcomes. Finally, caloric intake measurements obtained during hospitalization are inherently time-dependent, as their availability depends on patients remaining hospitalized. Consequently, analyses from later hospitalization days are subject to structural selection by LOS and cannot be interpreted as repeated longitudinal observations from a comparable cohort. Moreover, D2 caloric adequacy represents a post-baseline exposure measured during a period already included in total LOS, and feeding recovery may itself influence discharge decisions, resulting in potential outcome–exposure overlap.

## 5. Conclusions

In conclusion, lower estimated caloric adequacy during the first 48 h was associated with longer hospital LOS in children hospitalized with bronchiolitis. Failure to achieve at least 80% of estimated caloric requirements during this period was more frequent among patients with prolonged admissions, suggesting that early caloric adequacy may serve as a marker of clinical course. However, this association may partly reflect underlying disease severity, feeding tolerance, and clinical management, including discharge practices. The ≥80% threshold should not be interpreted as a therapeutic target, and prospective studies are needed to determine whether interventions aimed at improving early caloric intake can influence clinical outcomes.

## Figures and Tables

**Table 1:** Baseline characteristics of the study population.

Variable		Total Cohort (*n* = 613)
Male sex *n* (%)		366 (60)
Age at admission, days, median (IQR)		71 (34–124)
Prematurity, *n* (%)		61 (10)
Feeding at home	Formula feeding, *n* (%)	282 (46)
	Breastfeeding, *n* (%)	245 (40)
	Mixed feeding, *n* (%)	86 (14)
Feeding refusal before admission, *n* (%)		460 (75)
WDFM severity score at ED presentation, *n* (%)	Mild	107 (18)
	Moderate	452 (74)
	Severe	54 (9)
HSJD severity score at ED presentation, *n* (%)	Mild	236 (39)
	Moderate	370 (60)
	Severe	7 (1)
Supplemental oxygen therapy, *n* (%)		378 (62)
High-flow nasal cannula therapy, *n* (%)		28 (5)
PICU admission, *n* (%)		25 (4)
Hospital LOS, days, median (IQR)		5 (3–7)
Nutritional status at admission (WHO weight-for-length z-score) *	Severe acute malnutrition (z-score < −3 SD)	8 (1)
	Moderate acute malnutrition (z-score −3 to −2 SD)	40 (7)
	Normal nutritional status (z-score −2 to +1 SD)	488 (80)
	Risk of overweight (z-score +1 to +2 SD)	59 (10)
	Overweight (z-score +2 to +3 SD)	15 (2)
	Obesity (z-score > +3 SD)	0 (0)

Data are presented as median (interquartile range [IQR]) or *n* (%), as appropriate. Nutritional status was classified according to World Health Organization (WHO) weight-for-length z-scores [22]. * Nutritional status at admission was available for 610 patients; anthropometric data were unavailable for three patients. Abbreviations: IQR: interquartile range; WDFM: modified Wood–Downes–Ferrés score; ED: emergency department; HSJD: Hospital Sant Joan de Déu; PICU: pediatric intensive care unit; LOS: length of stay; WHO: World Health Organization; SD: standard deviation.

**Table 2:** Daily caloric adequacy according to hospital LOS.

Hospital LOS	Day 1	Day 2	Day 3	Day 4	Day 5	Day 6	Day 7	Day 8	Day 9
1 day (*n* = 11)	87 (75–97)	–	–	–	–	–	–	–	–
2 days (*n* = 62)	86 (73–97)	90 (76–102)	–	–	–	–	–	–	–
3 days (*n* = 92)	83 (66–95)	88 (79–105)	89 (75–105)	–	–	–	–	–	–
4 days (*n* = 107)	87 (72–97)	87 (77–102)	87 (77–102)	92 (85–105)	–	–	–	–	–
5 days (*n* = 81)	76 (64–102)	90 (79–103)	95 (79–105)	91 (78–104)	94 (79–105)	–	–	–	–
6 days (*n* = 86)	79 (60–93)	77 (66–94)	85 (71–103)	89 (79–103)	91 (79–105)	91 (82–103)	–	–	–
7 days (*n* = 72)	76 (53–89)	78 (64–94)	85 (74–94)	89 (76–98)	89 (77–102)	87 (77–99)	90 (78–103)	–	–
8 days (*n* = 30)	82 (65–92)	77 (69–102)	89 (76–98)	87 (73–100)	88 (79–105)	88 (76–105)	92 (82–105)	95 (88–105)	–
≥9 days (*n* = 72)	74 (63–88)	76 (62–89)	79 (60–89)	79 (63–92)	78 (64–97)	85 (69–95)	87 (76–99)	93 (78–102)	96 (84–108)

Values are expressed as median (IQR). The *n* values shown for each LOS category represent the total number of patients in that LOS group; caloric intake data were available for 607 patients overall. Daily caloric adequacy was defined as the percentage of age-specific caloric goals achieved according to WHO/FAO recommendations (% caloric adequacy/day) [19,20]. Breastfeeding caloric intake was estimated using a previously published probabilistic model, and the median estimate (50th percentile) was used for the main analyses [14]. Cells shaded in light red indicate caloric adequacy <80%, whereas cells shaded in light green indicate caloric adequacy ≥80%. Abbreviations: IQR: interquartile range; LOS: length of stay; WHO: World Health Organization; FAO: Food and Agriculture Organization.

**Table 3:** Correlation between daily caloric adequacy and hospital LOS, stratified by hospitalization day.

Hospitalization Day	*n*	Daily Caloric Adequacy According to the Medium Estimate, Median (IQR)	Correlation (r)	*p*-Value	Correlation (ρ)	*p*-Value
1	607	80 (65–94)	**−0.114**	**0.005**	**−0.150**	**<0.001**
2	594	86 (71–100)	**−0.126**	**0.002**	**−0.230**	**<0.001**
3	508	87 (74–102)	**−0.227**	**<0.001**	**−0.177**	**<0.001**
4	401	88 (76–102)	**−0.271**	**<0.001**	**−0.250**	**<0.001**
5	288	88 (76–103)	**−0.237**	**<0.001**	**−0.193**	**<0.001**
6	214	87 (76–101)	**−0.204**	**0.003**	**−0.175**	**0.011**
7	135	89 (76–102)	−0.120	0.166	−0.106	0.223
8	88	95 (83–105)	−0.088	0.412	−0.118	0.275
≥9	49	96 (84–108)	0.002	0.989	−0.212	0.144

Daily caloric adequacy was defined as the percentage of age-specific caloric goals achieved according to WHO/FAO recommendations [19,20]. For each hospitalization day, only patients with complete 24-h caloric intake data for that day were included. Caloric requirements were calculated using the medium breastfeeding estimate derived from the probabilistic breastfeeding model [14]. Abbreviations: IQR: interquartile range; LOS: length of stay; WHO: World Health Organization; FAO: Food and Agriculture Organization. Results are presented as Pearson correlation coefficients (r) and Spearman correlation coefficients (ρ). Bold values indicate statistically significant findings.

**Table 4:** Multivariable analyses of the association between day-2 caloric adequacy and hospital length of stay.

Model	D2 Exposure (% of ECR)	Severity Metric	Effect on LOS	95% CI	*p*
Primary	+10 pp	WDFM	−3.32%	−4.87, −1.74%	<0.001
Sensitivity (severity)	+10 pp	HSJD	−3.38%	−4.94, −1.79%	<0.001
Secondary (80% cut-point)	≥80% vs. <80% of ECR	WDFM	−18.68%	−26.77, −10.75%	<0.001
Landmark (48 h)	+10 pp	WDFM	−5.10%	−7.36, −2.79%	<0.001

All models were adjusted for age at admission, prematurity, feeding refusal, weight-for-length z-score at admission, home feeding modality, and study phase. The primary and severity sensitivity models estimated the percentage change in LOS associated with a 10-percentage-point increase in day-2 caloric adequacy. The secondary analysis compared patients achieving ≥80% versus <80% of estimated caloric requirements on day 2. The landmark analysis was anchored at the end of day 2 and estimated the percentage change in remaining LOS associated with a 10-percentage-point increase in day-2 caloric adequacy. Abbreviations: D2: hospitalization day 2; ECR: estimated caloric requirements; pp: percentage points; LOS: length of stay; WDFM: modified Wood–Downes–Ferrés score; HSJD: Hospital Sant Joan de Déu bronchiolitis severity score; CI: confidence interval.

**Table 5:** D2 caloric adequacy and its correlation with hospital LOS according to patient subgroups.

Variable		*n*	D2 Daily Caloric Adequacy, Median (IQR)	*p*-Value	Correlation (*r*)	*p*-Value	Correlation (ρ)	*p*-Value
Overall cohort		**594**	85 (70–99)	–	**−0.193**	**<0.001**	**−0.228**	**<0.001**
Age (months)	<6	498	85 (71–99)	0.357	−0.064	0.150	**−0.147**	**<0.001**
	6–11	67	82 (66–102)		**−0.281**	**0.020**	**−0.269**	**0.026**
	12–17	21	93 (69–111)		−0.318	0.149	−0.368	0.092
	18–24	8	69 (44–95)		0.073	0.851	0.050	0.898
Sex	Female	240	84 (68–98)	0.661	−0.113	0.078	**−0.183**	**0.004**
	Male	354	85 (71–100)		−0.077	0.144	**−0.153**	**0.003**
Feeding type at home	Breastfeeding	240	87 (76–102)	**0.035**	−0.113	0.079	**−0.182**	**0.004**
	Formula feeding	271	82 (67–97)		−0.044	0.465	**−0.121**	**0.045**
	Mixed feeding	83	83 (70–102)		−0.097	0.377	−0.204	0.062
Nutritional status at admission (WHO weight-for-length z score)	Severe acute malnutrition	8	89 (79–98)	0.602	−0.377	0.227	−0.096	0.766
	Moderate acute malnutrition	40	89 (70–105)		**−0.456**	**0.001**	**−0.449**	**0.002**
	Normal nutritional status	476	85 (69–99)		−0.083	0.069	**−0.171**	**<0.001**
	Risk of overweight	55	78 (68–91)		0.232	0.082	0.108	0.425
	Overweight	15	84 (70–97)		0.022	0.939	0.072	0.798

Daily caloric adequacy was defined as the percentage of age-specific caloric goals achieved according to WHO/FAO recommendations [19,20]. Caloric requirements were calculated using the medium breastfeeding estimate derived from the probabilistic breastfeeding model [14]. Nutritional status was classified according to World Health Organization (WHO) weight-for-length z-scores [22]. Abbreviations: D2: day 2; IQR: interquartile range; WHO: World Health Organization. Results are presented as Pearson correlation coefficients (r) and Spearman correlation coefficients (ρ). Bold values indicate statistically significant findings.

## Data Availability

The data presented in this study are not publicly available due to ethical and privacy restrictions involving patient data. De-identified data are available from the corresponding author upon reasonable request and with permission from the Ethics Committee.

## Abbreviations

The following abbreviations are used in this manuscript:

BF	Breastfeeding
CI	Confidence Interval
D2	Hospitalization Day 2
ECR	Estimated Caloric Requirements
FAO	Food and Agriculture Organization
HFNC	High-Flow Nasal Cannula
HSJD	Hospital Sant Joan de Déu
ICD-11	International Classification of Diseases, 11th Revision
IQR	Interquartile Range
LOS	Length of stay
PICU	Pediatric Intensive Care Unit
PP	Percentage Points
SD	Standard Deviation
SPSS	Statistical Package for the Social Sciences
WDFM	Modified Wood–Downes–Ferrés score
WHO	World Health Organization

## Appendix A

**Table A1:** Full multivariable regression models for the association between day-2 caloric adequacy and hospital length of stay.

Covariate	Primary β (*p*)	Sensitivity (HSJD) β (*p*)	Secondary (80%) β (*p*)	Landmark β (*p*)
D2 (scale per Table 4)	−0.0332 (<0.001)	−0.0338 (<0.001)	−0.1868 (<0.001)	−0.0524 (<0.001)
Age, days	−0.0003 (0.067)	−0.0003 (0.056)	−0.0004 (0.042)	−0.0003 (0.248)
Prematurity (ordinal)	−0.0212 (0.246)	−0.0205 (0.262)	−0.0200 (0.273)	−0.0390 (0.156)
Baseline severity (WDFM/HSJD)	0.0986 (0.013)	0.0864 (0.028)	0.1002 (0.011)	0.0812 (0.170)
Feeding refusal, partial (vs. no)	0.0696 (0.131)	0.0638 (0.166)	0.0785 (0.086)	0.0696 (0.316)
Feeding refusal, total (vs. no)	0.1866 (0.158)	0.1986 (0.133)	0.2064 (0.117)	0.1740 (0.356)
Weight/length z-score	−0.0155 (0.355)	−0.0153 (0.361)	−0.0192 (0.251)	−0.0257 (0.298)
Feeding, formula (vs. breastfeeding)	0.0281 (0.514)	0.0280 (0.517)	0.0220 (0.609)	0.1033 (0.109)
Feeding, mixed (vs. breastfeeding)	0.0229 (0.704)	0.0253 (0.675)	0.0035 (0.954)	0.1623 (0.076)
Study phase (prospective vs. retrospective)	−0.0523 (0.200)	−0.0454 (0.264)	−0.0432 (0.289)	−0.0487 (0.421)

β coefficients are presented on the log-transformed LOS scale for the primary, severity sensitivity, and secondary models, and on the log-transformed remaining LOS scale for the landmark analysis. The 95% CIs for the D2 caloric adequacy coefficients are reported in Table 4. Abbreviations: LOS: length of stay; D2: hospitalization day 2.

**Table A2:** Comparison of patient characteristics between the retrospective and prospective study phases.

Variable	Retrospective (*n* = 255)	Prospective (*n* = 358)	*p* (Raw)	*p* (Bonferroni, 11 Tests)
Age at admission, median [IQR]	56.0 [29.0–105.0]	78.0 [41.0–142.8]	<0.0001	0.0006
Sex (female/male)	112/143	135/223	0.144	1.000
Prematurity (0/1/2/3/4)	235/0/2/4/14	317/4/5/3/29	0.223	1.000
Feeding refusal (No/Partial/Total)	77/167/9	76/276/5	0.010	0.112
Feeding type (BF/Formula/Mixed)	104/116/35	143/163/51	0.857	1.000
Weight/length z-score, median [IQR]	−0.3 [−1.2, 0.4]	−0.4 [−1.1, 0.3]	0.816	1.000
WDFM severity (1/2/3)	52/192/11	55/260/43	0.002	0.023
HSJD severity (1/2/3)	110/142/3	126/228/4	0.134	1.000
D2 adequacy, %, median [IQR]	81.2 [65.9–92.6]	87.3 [74.2–103.1]	0.0007	0.0076
D2 missingness, *n* (%)	0 (0.0%)	3 (0.8%)	0.380	1.000
LOS, days, median [IQR]	5.0 [4.0–7.0]	5.0 [3.0–7.0]	0.073	0.798

Data are presented as median [IQR], *n*, or *n* (%), as appropriate. Raw *p*-values were adjusted for multiple comparisons using the Bonferroni correction (11 comparisons); adjusted *p*-values were calculated as raw *p* × 11 and capped at 1.00. Statistically significant Bonferroni-adjusted *p*-values (*p* < 0.05) are shown in bold. Abbreviations: BF: breastfeeding; D2: hospitalization day 2; WDFM: modified Wood–Downes–Ferrés score; HSJD: Hospital Sant Joan de Déu bronchiolitis severity score; LOS: length of stay; IQR: interquartile range.
